# The cellular activating protein-1 cFos regulates influenza A virus replication

**DOI:** 10.1099/jgv.0.002194

**Published:** 2026-01-14

**Authors:** Antoine Gerodez, François E. Dufrasne, Olivier Denis, Mieke Steensels, Bénédicte Lambrecht, Lionel Tafforeau, Caroline Demeret, Cyril Barbezange

**Affiliations:** 1 National Influenza Center, Sciensano, 1180 Brussels, Belgium; 2 Avian Virology and Immunology Service, Sciensano, 1180 Brussels, Belgium; 3 Immune Response Service, Sciensano, 1180 Brussels, Belgium; 4 Cell Biology laboratory, Research Institute for Health Sciences and Technologies, University of Mons, 7000 Mons, Belgium; 5 Interactomics, RNA and Immunity laboratory, Institut Pasteur, 75015 Paris, France

**Keywords:** AP-1, apoptosis, cFos, influenza A virus, interferon, transcription factor

## Abstract

Previous research has demonstrated that influenza A virus (IAV) infection activates activating protein-1 (AP-1) transcription factors as part of the antiviral response. In this study, we identified cFos as the most upregulated AP-1 transcription factor during IAV infection in A549 human lung cells. Surprisingly, the knockdown of cFos resulted in impaired IAV replication. Fluorescence microscopy and functional analyses indicated that cFos is implicated in IAV infection through its nuclear function, rather than its cytoplasmic role as an activator of lipid synthesis. The investigation into the role of cFos in IAV infection revealed increased apoptosis and elevated interferon-*β* mRNA levels in cFos–knockdown A549 cells during IAV infection. This suggests that cFos may enhance cell survival and reduce interferon-*β* expression during infection, thereby facilitating IAV proliferation. Furthermore, the levels of viral NA mRNA and the expression of late viral proteins NA and M2 decreased upon cFos–knockdown. Overall, this study identifies cFos as a proviral factor for IAV, through the modulation of innate immunity and apoptosis during infection, and potentially by supporting the viral transcription.

## Data Summary

The raw data used to produce the figures as reported in the manuscript were deposited in figshare.com in two sets: https://doi.org/10.6084/m9.figshare.29880887 and https://doi.org/10.6084/m9.figshare.29880806.

## Introduction

Activating protein-1 (AP-1) transcription factors consist of homo- and hetero-dimers composed of basic region-leucine zipper proteins that belong to Jun (cJun, JunB and JunD), Fos (cFos, FosB, Fra-1 and Fra-2), Maf (c-Maf, MafB, MafA, MafG/F/K and Nrl) and ATF (ATF2, LRF1/ATF3, B-ATF, JDP1 and JDP2) families. These transcription factors regulate many cellular functions, including cell proliferation, differentiation, inflammation and apoptosis (reviewed in refs. [1,2]). cFos and cJun are commonly associated in a complex that binds in a sequence-specific manner to the promoter and enhancer regions of target genes [3].

Although Jun and Fos families have been considered to regulate cell proliferation positively (reviewed in ref. [4]), the role of cFos in cell proliferation remains debatable. While mouse fibroblasts deficient in both cFos and FosB had a reduced proliferative activity, the inhibition of cFos alone induced no effect on cell [5]. In addition, double-knockout mice lacking both cFos and FosB, but not the single knockout mice, had smaller body sizes than their wild-type counterparts [6]. On the other hand, the knockdown of cFos resulted in inhibited proliferation of a human osteosarcoma cell line, and the stable overexpression of cFos led to increased proliferation of immortalized human hepatocytes under low serum conditions [7,8]. cFos also regulates apoptosis. Although cFos was shown to promote apoptosis in different cell types [9,11], it seemed to repress apoptosis in a human osteosarcoma cell line and to decrease neuronal cell death in the hippocampus during kainic acid-induced seizure [7,12]. Therefore, the regulation of the cell fate by cFos and the AP-1, in general, is complex and depends on the cell type, the type and duration of the stimulus and the involvement of other transcription factors. In addition, AP-1 has been described as an activator of inflammation (reviewed in ref. [2]), although some evidence also suggests a specific anti-inflammatory role of cFos. A significant increase in the pro-inflammatory cytokines such as TNF-*α*, IL-6 and IL-12 p40 was observed in mouse macrophages and mice deficient in cFos [13], while the expression of cFos was observed to inhibit IL-12 p40 promoter activity in mouse macrophages [14]. Finally, independently of its transcriptional factor activity in the nucleus, cFos possesses a cytoplasmic function as an activator of lipid synthesis at the endoplasmic reticulum level (reviewed in ref [15]).

Several viruses were shown to hijack AP-1 proteins to support their replication. AP-1 binding to the intragenic regulatory region of the *pol* gene of the human immunodeficiency virus type 1 seemed to help recruit the cellular DNA-dependent RNA-polymerase II to the viral promoter, supporting viral transcription [16]. cFos was found to bind to multiple gene promoters of Kaposi’s sarcoma-associated herpesvirus and to enhance viral lytic transcription [17]. siRNA-based experiment identified a role of cFos in hepatitis C virus replication and propagation [18]. cFos was also shown to promote virus replication of alpha- and gamma-coronaviruses by delaying and reducing apoptosis [19].

Influenza A viruses (IAVs) are major respiratory pathogens responsible for human seasonal epidemics and pandemics, posing a persistent threat to global health. IAVs are enveloped viruses and belong to the *Orthomyxoviridae* family [20]. Their genome consists of eight negative-sense single-stranded RNA segments. Each viral RNA (vRNA) segment is encapsidated by nucleoproteins (NP) and attached to the heterotrimeric polymerase complex, composed of the PB1, PB2 and PA subunits, thus forming the viral ribonucleoprotein (vRNP). To start an infection, IAV enters the cell through an endocytic pathway. Inside the cell, vRNPs are released and transported to the nucleus, where viral transcription and replication occur. Transcription and replication are carried out by the viral polymerase complex. Primary transcription generates viral mRNAs, which are exported to the cytoplasm for translation by host ribosomes. PB2, PB1, PA, NP and NS1 are expressed early, while HA, NA, M1 and NS2 are expressed later during the viral cycle [21]. Viral proteins PB2, PB1, PA, NP, M1 and NS2 are transported back to the nucleus, and genome replication then occurs. Newly synthesized vRNAs are assembled with PB2, PB1, PA and NP, resulting in progeny vRNPs that are subsequently exported to the cytoplasm. These vRNPs are further incorporated into progeny particles containing HA, NA, M2 and M1 inserted in or present at the cell membrane. Finally, progeny virions are released from the cell by budding for subsequent infection [20,22].

AP-1 transcription factors might play a role in IAV polymerase activity regulation, as the inhibition of cJun was shown to impair IAV H5N1 replication in human lung cells [23]. However, AP-1 also appears to take part in the innate antiviral response following IAV infection. IAV-induced AP-1 activation was shown to activate the expression of interferon-*β* and promote NLRP3 inflammasome activation [24,25]. Furthermore, the viral protein NS1 antagonized IAV-induced AP-1 activation [26]. Thus, the role of the AP-1 transcription factors in IAV infection remains unclear. In this study, we observed that the infection of human cells with human IAVs resulted in the upregulation of cFos and cJun subunits. The role of cFos was then investigated using specifically depleted cells, showing that knockdown significantly impaired viral replication efficiency. Further characterization revealed different potential mechanisms by which cFos may support IAV replication.

## Methods

### Cell lines

HEK293T, A549, MDCK and MDCK-SIAT1 [27] cells were provided by Institut Pasteur Paris. HEK293T and A549 cells were grown in Dulbecco’s Modified Eagle’s Medium (DMEM) (Life Technologies cat#41965039) supplemented with 10% FBS (Life Technologies cat#10270-106), 10 U ml^−1^ of penicillin and 10 µg ml^−1^ of streptomycin (Life Technologies cat#15140122). MDCK and MDCK-SIAT1 cells were grown in Modified Eagle’s Medium (Life Technologies cat#31095029) supplemented with 5% FBS, 10 U ml^−1^ of penicillin and 10 µg ml^−1^ of streptomycin. All cell lines were grown at 37 °C in 5% CO_2_.

### Viruses

Three human IAVs were used: the two currently circulating seasonal IAVs in the human population, A/Bretagne/7608/2009 (H1N1)pdm09 (referred to as pH1N1 in ‘Results’) and A/Centre/1003/2012 (H3N2) and the laboratory-adapted A/WSN/33 (H1N1) (referred to as WSN in ‘Results’). All viruses were produced by reverse genetics [28,29] and amplified on MDCK cells, except for the H3N2 strain, which was amplified on MDCK-SIAT1 cells. pH1N1 and H3N2 subtypes grow poorly in A549 cells. Two mutations in the pH1N1 HA (A517G and G834A) and one mutation in the H3N2 HA (G460T) were introduced by site-directed mutagenesis into the HA-encoding plasmid pH1N1 and H3N2 of the reverse genetic system to generate A549-adapted pH1N1 and H3N2 strains, capable of efficient replication in A549 cells [30]. Four avian IAVs were used (Fig. S1, available in the online Supplementary Material): A/Anas platyrhynchos/Belgium/14325/07 (H3N8); A/Anas platyrhynchos/Belgium/10399/2018 (H4N6); A/Gallus_gallus/Belgium/ 16070_0002/2021 (H5N1); and A/chicken/Israel/1163/2011 (H9N2). All avian IAVs were initially isolated in embryonated chicken eggs, except for H9N2, which was isolated on MDCK cells. All avian IAVs were subsequently propagated in MDCK cells for experimental use.

### Antibodies, chemicals and reagents

Rabbit anti-*β*-actin (cat#4967), rabbit anti-cFos (cat#2250), goat anti-rabbit IgG, HRP-linked (cat#7074) and horse anti-mouse IgG, HRP-linked (cat#7076) antibodies were purchased from Cell Signaling Technology. Mouse anti-M2 (cat#MA1-082), rabbit anti-NP (cat#PA5-32242), chicken anti-calreticulin (cat#PA1-902A), chicken anti-fibrillarin (cat#PA5-143565A), goat anti-rabbit Alexa Fluor Plus 647 (cat#A32733) and goat anti-chicken IgY (H+L) Alexa Fluor 594 (cat#A-11042) antibodies were purchased from Thermo Fisher Scientific. The rabbit anti-NA antibody (cat#GTX125974) was purchased from Genetex. The mouse anti-NS1 (cat#sc-130568) and the mouse anti-NP (cat#sc-80481) antibodies were purchased from Santa Cruz Biotechnology. The goat anti-mouse IgG-FITC antibody (cat#F0257) was purchased from Sigma-Aldrich. The Annexin V-Alexa Fluor 488 apoptosis detection reagent (cat#A13201) was purchased from Thermo Fisher Scientific, and the 7-Amino-Actinomycin D (7-AAD) (cat#559925) was purchased from BD Biosciences. The T-5224 small inhibitor (cat#HY-12270) was purchased from MedChemExpress. CellTiter-Glo® Luminescent Cell Viability Assay (cat#G7570), Dual-Glo® Luciferase Assay System (cat#E2920) and *Renilla* Luciferase Assay System (cat#E2810) were purchased from Promega.

### siRNA-based assays

#### Multi-cycle infection

siRNAs were purchased from Horizon Discovery (ON-TARGETplus SMARTpools and non-targeting Control pool). The siRNA sequences were designed by Horizon Discovery and are listed in Table S1. 6.5 µl of siRNA at a concentration of 5 µM was added to 243.5 µl of DharmaFECT1 transfection reagent (Horizon discovery cat#T-2001) and OptiMEM GlutaMAX medium (Life Technologies cat#51985034) to obtain a final volume of 250 µl. The 250 µl mixture was added to one well of a 12-well tissue culture plate (Greiner). Following a 30-min incubation period at room temperature, 2×10^5^ A549 cells diluted in 1 ml of DMEM supplemented with 5% FBS were seeded on top of siRNA-transfection reagent complexes and incubated at 37 °C in 5% CO_2_. The final concentration of siRNA was 25 nM per well. At 48 h post-transfection (hpt), the cells were washed and infected with pH1N1 at a multiplicity of infection (moi) of 10^−2^ or 10^−3^, WSN at a moi of 10^−4^ and H3N2 at a moi of 10^−1^ or 10^−2^ at 35 °C in 5 % CO_2_. Supernatants were collected at 0, 24 and/or 48 h post-infection (hpi), and viral titres were determined by plaque assays using MDCK-SIAT1 cells [31].

#### Single-cycle infection

The cells were treated with siRNAs as described above. At 48 hpt, the cells were washed, inoculated with WSN at a moi of 3 and incubated for 1 h at room temperature. Inoculum was then removed, fresh OptiMEM GlutaMAX medium was added and cells were incubated at 35 °C (5% CO_2_). Timepoint 0 hpi was defined as the start of the cell incubation at 35 °C.

#### Cell viability and luciferase-based knockdown efficiency experiments

siRNA reverse transfection was performed in a white 96-well tissue culture plate (Greiner). Volumes were adjusted to obtain a siRNA final concentration of 25 nM per well. Briefly, 0.65 µl of siRNA at a concentration of 5 µM was added to 24.35 µl of DharmaFECT1 transfection reagent and OptiMEM GlutaMAX medium to obtain a final volume of 25 µl. The 25 µl mixture of siRNA-DharmaFECT1 was added to one well of the white 96-well plate. Following a 30-min incubation period at room temperature, 1.5×10^4^ A549 cells diluted in 100 µl of DMEM supplemented with 5% FBS were seeded on top of the siRNA-transfection reagent complexes and incubated at 37 °C in 5% CO_2_. Cell viability was determined at 48 hpt using the CellTiter-Glo Luminescent Viability Assay kit according to the manufacturer’s instructions (Promega). To measure the efficiency of siRNA knockdown, siRNA-treated A549 cells were transfected at 24 h post-siRNA transfection with 10 ng of plasmids encoding siRNA-targeted protein fused with the full-length Gaussia luciferase (pGlucFL) using Lipofectamine 3000 (Thermo Fisher Scientific cat#L3000001). The luciferase activity was measured 24 h later in cell lysates using the *Renilla* luciferase assay reagent (Promega). All luciferase activities were measured on a GloMax Explorer (Promega).

### Real-time quantitative PCR

RNAs were extracted using the Quick-RNA Miniprep Kit (Zymo Research cat#R1054) from A549 cells treated with siRNA and infected with WSN or mock-infected. The kit uses a column to remove most of the genomic DNA and a subsequent treatment with DNase I to degrade the remaining genomic DNA. RNA concentrations were measured with NanoDrop One (Thermo Fischer Scientific) and adjusted to 100 ng µl^−1^. Two hundred nanogram RNA was reverse transcribed using the Accuscript High Fidelity first-strand cDNA synthesis kit (Agilent cat#200820) following the manufacturer’s instructions. Briefly, 200 µg RNA was mixed with 4 µl buffer 10 × AccuScript High Fidelity RT-PCR System, 1.6 µl 100 mM dNTPs, 1 µg anchored-oligo(dT) primer, and nuclease-free water in a 33 µL reaction mixture. The mixture was heated for 5 min at 65 °C and cooled for 5 min to room temperature before the addition of 40 U RNase block, 10 mM DTT and 2 µl Accuscript RT in a 40 µl final volume reaction mixture. Reverse transcription was performed at 42 °C for 60 min in a thermocycler, and the RT enzyme was then inactivated at 72 °C for 15 min. Real-time quantitative PCR (qPCR) was performed using GoTaq qPCR master mix containing BRYT green dye (Promega cat#A6001) on an ARIA MX (Stratagene MX3005P, Agilent Technologies) in a total volume of 20 µl. Each reaction mixture included 10 µl GoTaq qPCR MasterMix, 1 µl of each diluted forward and reverse primers (10 µM), 1.25 µl template cDNA and nuclease-free water up to 20 µl. Primers for the AP-1 transcription factors (sequences based on ref. [19]) were purchased from Integrated DNA Technologies. Primer sequences for cytokine genes and phospholipid enzymes were designed and verified by Sigma-Aldrich. All the primers are listed in Table S2. A control with 1.25 µl of water instead of cDNA was included in each run to check for reagent contamination. A ‘No RT’ control was also included for each targeted gene of the real-time qPCR experiments to check the efficiency of the DNase I treatment. The thermal cycling conditions for all qPCRs were as follows: denaturation at 95 °C for 2 min, followed by 40 cycles consisting of 95 °C for 15 s, and an annealing and extension phase at 60 °C for 1 min. Afterwards, a melting curve analysis was performed to determine the specificity of the reaction products. The modulation of RNA expression for all targeted genes was normalized to *β*-actin expression (housekeeping gene) and analysed using the 2^-ΔΔCt^ method (delta-delta Ct method).

### Strand-specific real-time qPCR

RNAs were extracted using the Quick-RNA Miniprep Kit (Zymo Research cat#R1054) from A549 cells treated with siRNA and infected with WSN or mock-infected. Strand-specific real-time qPCR for NP and NA vRNAs, cRNAs and mRNAs was performed as previously described [32]. Briefly, cDNAs complementary to NP and NA vRNAs, cRNAs and mRNAs were synthesized using SuperScript™ IV Reverse Transcriptase (Thermo Fisher Scientific cat#18090010) and tagged primers in order to add a strand-specific tag unrelated to influenza virus sequence at the 5′ end. Tagged cDNAs were then used as a template for the qPCR reaction using a tag-specific primer and a segment-specific primer. *β*-Actin (housekeeping gene) mRNA was reverse transcribed using anchored-oligo(dT) and SuperScript™ IV Reverse Transcriptase (Thermo Fisher Scientific cat#18090010). All qPCRs were performed using GoTaq qPCR master mix containing BRYT green dye (Promega cat#A6001) on an ARIA MX (Stratagene MX3005P, Agilent Technologies) in a total volume of 20 µl. Each reaction mixture included 10 µl GoTaq qPCR MasterMix, 1 µl of each diluted forward and reverse primers (10 µM), 7 µl tenfold diluted template cDNA and nuclease-free water up to 20 µl. The primers were designed as described in ref. [32] to specifically detect NP and NA vRNAs, cRNAs and mRNAs. A control with 7 µl of water instead of cDNA was included in each run to check for reagent contamination. The thermal cycling conditions for all qPCRs were as follows: denaturation at 95 °C for 2 min, followed by 50 cycles consisting of 95 °C for 15 s, and an annealing and extension phase at 60 °C for 1 min. Afterwards, a melting curve analysis was performed to determine the specificity of the reaction products. The modulation of vRNA, cRNA and mRNA expression for all targeted genes was normalized to *β*-actin expression (housekeeping gene), and RNA fold changes relative to the condition 0 hpi were calculated using the 2^-ΔΔCt^ method. All the primers for both reverse transcription and qPCR are listed in Table S3.

### Western blot

A549 cells treated with siRNA and infected with WSN or mock-infected were lysed at 0, 3, 6 and 9 hpi using RIPA lysis and extraction buffer (Thermo Fisher Scientific cat#89900) supplemented with 100-fold diluted Halt™ Protease and Phosphatase Inhibitor Cocktail, EDTA-free (Thermo Fisher Scientific cat#78441). Briefly, the cells were washed with cold PBS, and 200 µl supplemented RIPA buffer was added to each well of the 12-well plate. Following a 15-min incubation period on ice, lysed cells were collected and centrifuged at 14,000 ***g*** for 15 min. Cell lysate supernatants were kept, and protein quantification was performed using Pierce™ BCA Protein Assay Kits (Thermo Fisher Scientific cat#23225) following the manufacturer’s instructions. Cell lysates were then mixed with 4× Laemmli Sample Buffer (BioRad cat#1610747) supplemented with 355 mM of *β*-mercaptoethanol (Sigma-Aldrich cat#M6250) and boiled at 90 °C for 10 min. Equal amounts of protein samples were loaded into each well and separated using 8–16% Mini-PROTEAN® TGX Stain-Free™ Protein Gel (BioRad cat#4568105) in a Mini-PROTEAN Tetra Vertical Electrophoresis Cell (BioRad cat#1658004), at 200 V for 30 min. The resolved proteins were then transferred to a 0.2 µM nitrocellulose membrane for 7 min at 25 V using the Trans-Blot® Turbo™ Transfer System (BioRad cat#1704150). The membrane was blocked for 1 h at room temperature with PBS-0.05% Tween 20–3 % BSA and then incubated with the indicated primary antibodies diluted in PBS-0.05% Tween 20–3 % BSA at 4 °C overnight. After 3 washes for 5 min with PBS-0.1% Tween 20, the membrane was incubated with 1:5,000 HRP-linked goat anti-rabbit IgG or HRP-linked horse anti-mouse IgG, at room temperature for 2 h. After 3 washes for 5 min with PBS-0.1% Tween 20, proteins were detected by chemiluminescence using Pierce™ ECL Plus Western Blotting Substrate (Thermo Fisher Scientific cat#32132) and ChemiDoc imaging system (Biorad). Protein band intensities were determined using ImageJ software. All experiments were repeated three times, with similar results, and one of the representative immunoblots is shown.

### Immunofluorescence staining

A549 (2×10^5^) cells were seeded on coverslips (13 mm in diameter) in a 12-well plate (Greiner) in DMEM supplemented with 10% FBS, 10 U ml^−1^ of penicillin and 10 µg ml^−1^ streptomycin and incubated at 37 °C for 24 h. Cells were infected with WSN at a moi of 3 p.f.u. cell^−1^. At 0, 3, 6 and 9 hpi, cells were fixed with 3% paraformaldehyde for 20 min and permeabilized with PBS-0.1% Triton X-100 for 20 min. Cells were blocked with PBS-3% BSA for 1 h and incubated with primary antibodies anti-cFos (1/200), anti-NP (1/200) and anti-fibrillarin (1/200) or anti-calreticulin (1/100) overnight at 4 °C. Following multiple washes with PBS, cells were incubated with FITC goat anti-mouse IgG (1/200), Alexa Fluor 594 goat anti-chicken IgY (1/200) and Alexa Fluor 647 goat anti-rabbit (1/300) secondary antibodies for 1 h at room temperature. Following multiple washes with PBS, coverslips were mounted in ProLong Gold Antifade Mountant with DNA Stain DAPI (Thermo Fisher Scientific cat#P36941) and analysed under an inverted fluorescence microscope (Nikon ECLIPSE Ts2R) using an ×60 objective lens. The percentage of nuclear signal for cFos was determined using the Fiji software. For each image, five infected cells were selected. For each cell, the integrated density of the red fluorescence, corresponding to cFos signal, was measured in the nucleus (defined by DAPI staining) and in the cytoplasm. The percentage of nuclear signal was calculated as the ratio of the integrated density in the nucleus to the total integrated density of the cell (nuclear+cytoplasmic).

### Minigenome assay

HEK293T (3×10^4^) cells were transfected with NT or cFos siRNA in a white 96-well tissue culture plate, as described above. After 48 h of knockdown, cells were transfected using Polyethyleneimine ‘MAX’ (MW 40,000) 1 mg ml^−1^ (Polysciences cat#24765) with expression pCIneo or pcDNA3 plasmid vectors encoding the IAV proteins PA, PB1, PB2 (25 ng each plasmid) and NP (50 ng) from different IAV strains (pH1N1, WSN and H3N2), a reporter plasmid vector (pPR7-firefly-(-)) encoding the firefly luciferase in the negative-sense orientation flanked by the noncoding regions of the segment 5 of WSN driven by a polymerase I promoter (5 ng) [33] and a plasmid vector (polIII-*Renilla*) constitutively expressing *Renilla* luciferase (5 ng). Twenty-four hours post-transfection, luciferase activities were measured using the Dual-Glo luciferase assay system (Promega). Polymerase activity, proportional to Firefly luciferase activity, was normalized to *Renilla* luciferase activity to take into account the transfection rate.

### Measure of apoptosis and necrosis rates

A549 cells were transfected with NT or cFos siRNA as described above. After 48 h of knockdown, cells were infected with WSN at a moi of 3 p.f.u. cell^−1^ or mock-infected. At 24 hpi, the apoptosis and necrosis rates were determined. Cells were washed with PBS and detached using trypsin-EDTA 0.05% phenol red (Thermo Fisher Scientific cat#25300054). DMEM supplemented with 10% FBS was then added, and the collected cells were centrifuged at 2,000 r.p.m. for 5 min. Pellet cells were resuspended in Annexin-binding buffer (HEPES 10 mM, NaCl 140 mM, CaCl_2_ 2.5 mM, pH 7.4) at 10^6^ cells ml^−1^. One hundred microlitres of cells was then transferred into a FACS tube, and 5 µl of Annexin V-Alexa Fluor 488 apoptosis detection reagent and 7-AAD was added. Staining was performed for 15 min at room temperature. Four hundred microlitres of Annexin-binding buffer was then added. Cells were further fixed in 4% paraformaldehyde at 4 °C in the dark until analysis. As a positive control, cells were also treated with 10 µM Camptothecin (Thermo Fisher Scientific cat#J62523.MD), known to induce apoptosis. Cells were acquired on a FACSVerse flow cytometer (BD Biosciences). Data were analysed with the FACSuite software (BD Biosciences). Around 2,000 events were counted for each sample.

### Statistical analysis

Unpaired Student’s t-test was used for all statistical analyses, using GraphPad Prism software v.9.00 (GraphPad Software). Differences between groups were considered statistically significant at *P*<0.05; significance levels are as follows: *P<0.05, **P<0.01, ***P<0.001, ns: non-significant.

## Results

### IAV multiplication upregulates the AP-1 transcription factors and is impaired in cFos–knockdown cells

**Fig. 1.**
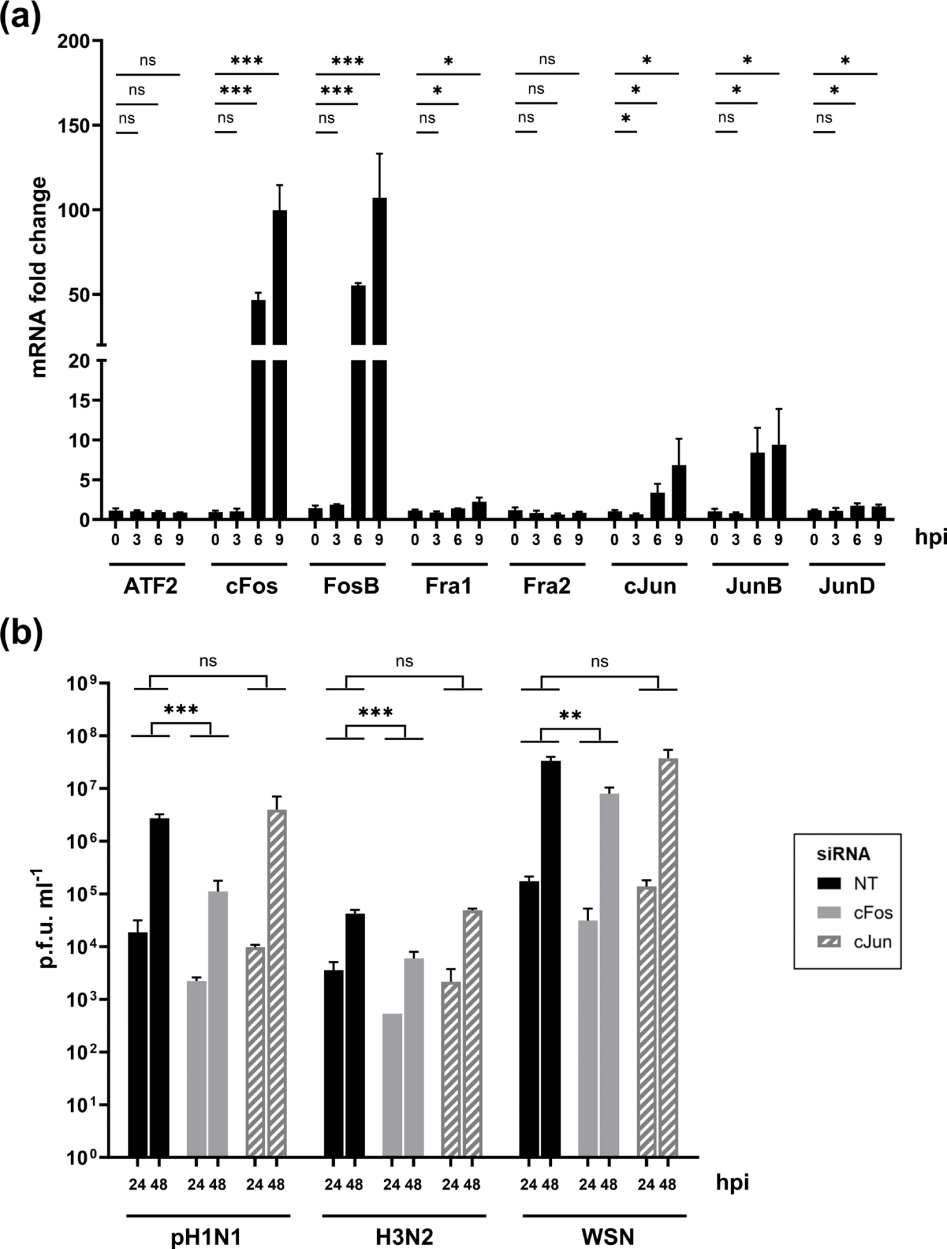
cFos mRNA expression is upregulated during IAV infection, and its knockdown reduced IAV replication. (**a**) A549 cells were infected with WSN at moi of 3 p.f.u. cell^−1^. Total RNAs were extracted at the indicated time point post-infection, and the mRNA expression levels of AP-1 were determined by real-time qPCR. mRNA fold changes were calculated using the 2^-ΔΔCt^ method compared to the mock cells at each time point. Results are expressed as the mean+sd determined in three independent experiments. The significance of the difference to the 0 hpi timepoint was tested with unpaired t-tests using GraphPad Prism software for each cellular factor (ns, not significant; *P<0.05, **P<0.01, ***P<0.001). (**b**) A549 cells were transfected with 25 nM of the NT or the indicated siRNAs. At 48 hpt, cells were infected with the following viruses at the indicated moi in p.f.u. cell^−1^: A/Bretagne/7608/2009(H1N1pdm09) (pH1N1, moi of 10^−3^); A/Centre/1003/2012(H3N2) (H3N2, moi of 10^−2^); A/WSN/33(H1N1) (WSN, moi of 10^−4^). At 0, 24 and 48 hpi, viral titres were determined by plaque‐forming assay. Results are expressed as the mean±sd p.f.u. ml^−1^ of three independent experiments. The area under the curve (AUC) (not shown) was determined for each virus and condition, taking the p.f.u. ml^−1^ at timing 0 hpi as the baseline. The significance was tested on AUCs with unpaired t-tests using GraphPad Prism software (ns, not significant; *P<0.05, **P<0.01, ***P<0.001).

Following single-cycle IAV infection in A549 cells, the expression of AP-1 transcription factors cFos, FosB, cJun and JunD was upregulated at 6 and 9 hpi, as measured by real-time qPCR. The mRNA level of cFos and FosB was >50-fold higher than in mock-infected cells. For cJun and JunB, the mRNA level at 9 hpi was between 5 and 10 times higher in infected cells. No difference in the mRNA expression of Fra1, Fra2, JunD and ATF2 was observed at any time point (Fig. 1a). The role of cFos and cJun on viral replication was further investigated using small interfering RNA (siRNA)-mediated silencing. Upon individual siRNA knockdown of cFos and cJun, A549 cell viability remained above 90% compared to the non-target (NT) siRNA-treated cells, with knockdown efficiency of protein expression estimated at 92% and 61% for cFos and cJun, respectively (Fig. S1a, b). Human IAV replication was not affected by the individual knockdown of cJun (Fig. 1b). In contrast, cFos–knockdown significantly impaired the replication of human seasonal pH1N1, H3N2 and WSN IAV strains (Fig. 1b). The effect was also observed for IAVs of avian origin (Fig. S1d). Knockdown of the well-described proviral factor RAB11 [34] was used as a control (Fig. S1c).

### The nuclear function of cFos, but not its cytoplasmic function, regulates IAV multiplication

**Fig. 2.**
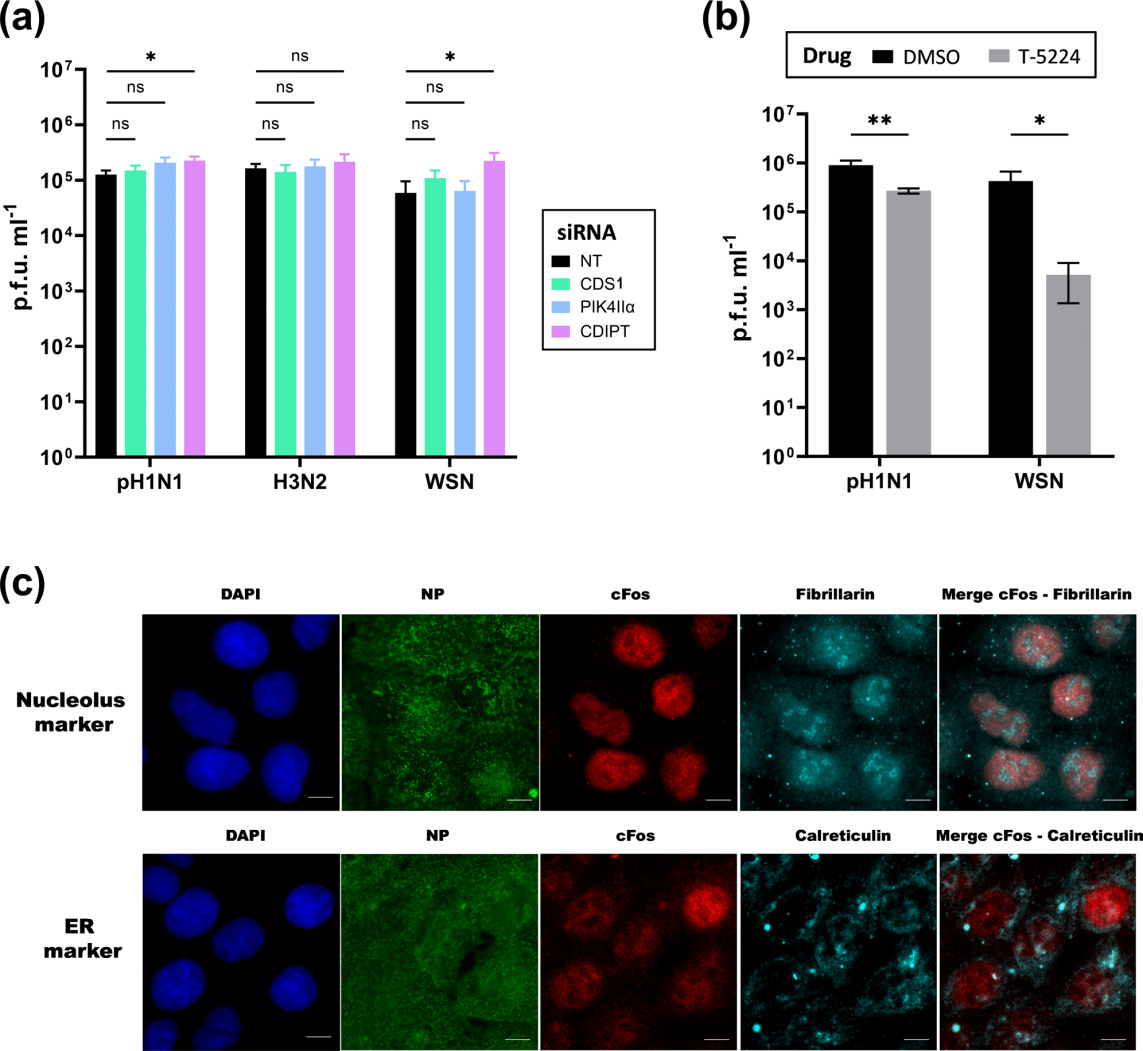
The nuclear function of cFos, but not its cytoplasmic function as an activator of lipid synthesis, appears to regulate IAV multiplication. (**a**) A549 cells were transfected with 25 nM of the NT or the indicated siRNAs. At 48 hpt, cells were infected with the following viruses at the indicated moi in p.f.u. cell^−1^: pH1N1, moi of 10^−2^; H3N2, moi of 10^−1^; and WSN, moi of 10^−4^. At 24 hpi, viral titres were determined by plaque‐forming assay. Results are expressed as the mean±sd of three independent experiments, and the significance was tested with multiple unpaired t-tests using GraphPad Prism software (ns, not significant, *P<0.05). (**b**) A549 cells were treated with DMSO (black bars) or 20 µM T5224 (grey bars). At 3 h post-treatment, the cells were infected with the following viruses at the indicated moi in p.f.u. cell^−1^: pH1N1, moi of 10^−2^, and WSN, moi of 10^−4^ in the presence of DMSO or T5224 20 µM. At 24 hpi, viral titres were determined by plaque‐forming assay. Results are expressed as the mean±sd of three independent experiments, and the significance of the difference to NT was tested with multiple unpaired t-tests using GraphPad Prism software for each virus (*P<0.05, **P<0.01). (**c**) Immunofluorescence staining of cFos, fibrillarin (nucleolus marker) and calreticulin (ER marker) in infected A549 cells (WSN, moi of 3). Cells were fixed at 6 hpi, stained with DAPI and immunostained with anti-NP (infection control), anti-cFos and anti-fibrillarin or anti-calreticulin. Scale bar=10 µm.

We were further interested in deciphering which function of cFos could support IAV replication. At the endoplasmic reticulum (ER), cFos activates phospholipid synthesis by physically interacting with the CDP diacylglycerol synthase 1 (CDS1) and phosphatidylinositol 4 kinase type II*α* (PI4KII*α*) enzymes of the polyphosphoinositide (PIP) lipid pathway [15]. On the contrary, another enzyme involved in the PIP pathway, the CDP diacylglycerol inositol 3 phosphatidyltransferase enzyme, is not regulated by cFos [15]. No major differences in IAV replication were detected upon siRNA-based knockdown of both cFos-activated enzymes CDS1 and PI4KII*α* compared to NT siRNA-treated A549 cells (Fig. 2a), with cell viability and knockdown efficiency remaining above 95% (Fig. S2). In contrast, upon treatment with T-5224, a specific inhibitor of cFos/AP-1 DNA binding [35], IAV replication was impaired. The viral titres for pH1N1 and WSN viruses were significantly lowered by 0.5 and 2 log_10_, respectively, in A549 cells treated with 20 µM T-5224 compared with DMSO-treated cells (Fig. 2b), with cell viability remaining superior to 50% (Fig. S3a). At 6 hpi, cFos was mainly localized in the nucleus, more specifically in the nucleoplasm but not in the nucleoli (marked by anti-fibrillarin), and hardly in the ER (marked by anti-calreticulin) (Figs 2c and S4).

### The apoptosis is increased in cFos–knockdown cells during IAV infection

**Fig. 3.**
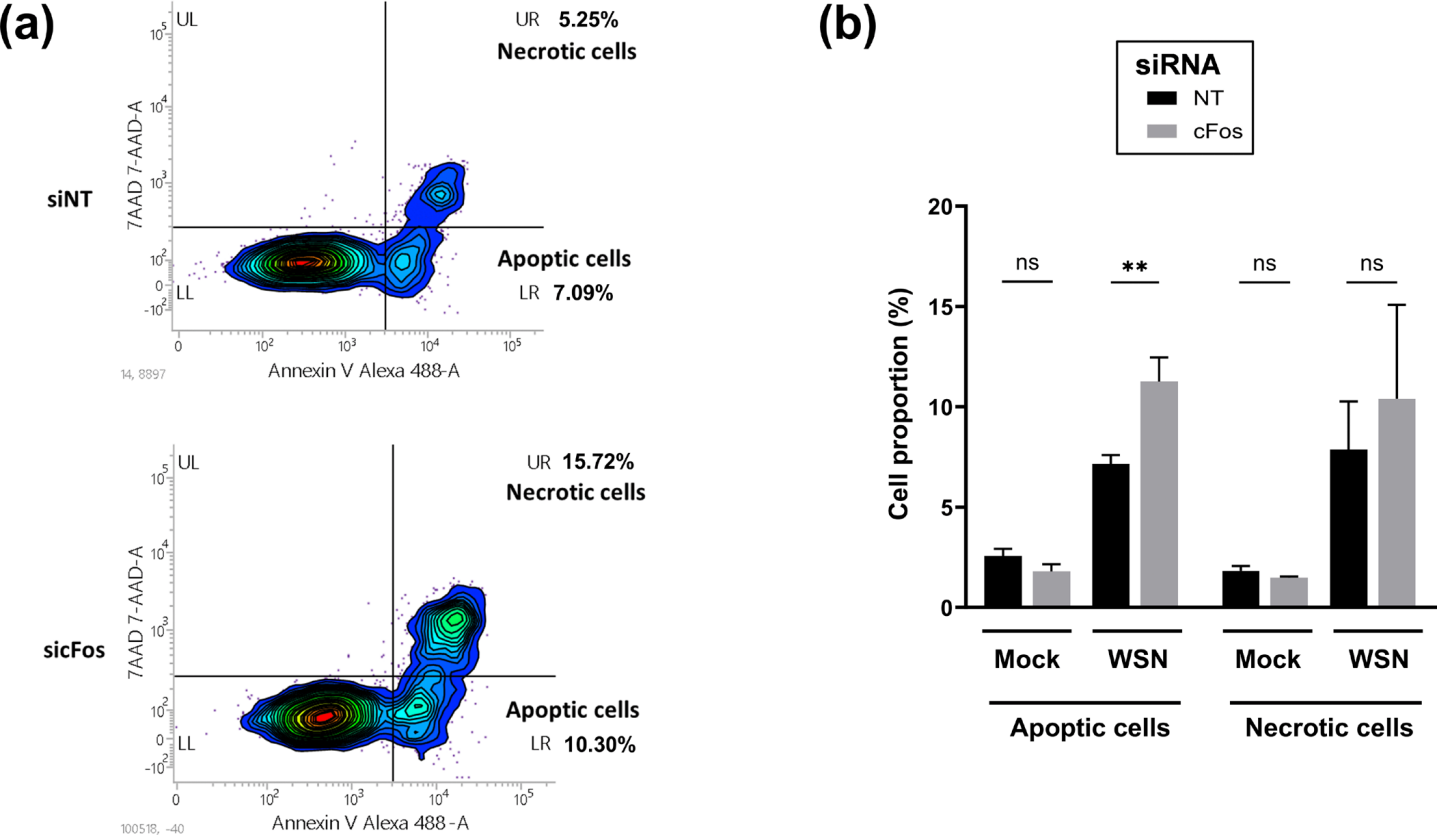
Apoptosis is increased in cFos–knockdown cells during IAV infection. A549 cells were treated with 25 nM of the NT or cFos siRNAs and infected 48 hpt with WSN at a moi of 3 p.f.u. cell^−1^. At 24 hpi, cells were stained with 7AAD (necrotic cells) and Annexin V-AlexaFLuor 488 (apoptotic cells) and subjected to flow cytometry analysis (around 2000 cells analysed). Mock cells were included in the experiment. (**a**) Contour plot of infected cells treated with NT or cFos siRNA, representative of three independent experiments. UR, necrotic cells; LR, apoptotic cells. (**b**) Relative quantification was performed for necrotic and apoptotic cells. Results are expressed as the mean±sd of three independent experiments, and the significance of the difference to NT was tested with multiple unpaired t-tests using GraphPad Prism software (ns, not significant; **P<0.01).

cFos regulates apoptosis via its nuclear activity [9,10]. In non-infected cells, upon cFos–knockdown or in NT control cells, apoptosis and necrosis rates were about 2% (Fig. 3b). Upon IAV infection, both apoptosis and necrosis increased (Fig. 3a and b). The apoptosis rate was significantly higher in cFos–knockdown cells (12%) compared to control cells (7%). A similar trend was also observed with necrosis, but the difference was not statistically significant (Fig. 3a and b).

### cFos regulates inflammation during IAV infection

**Fig. 4.**
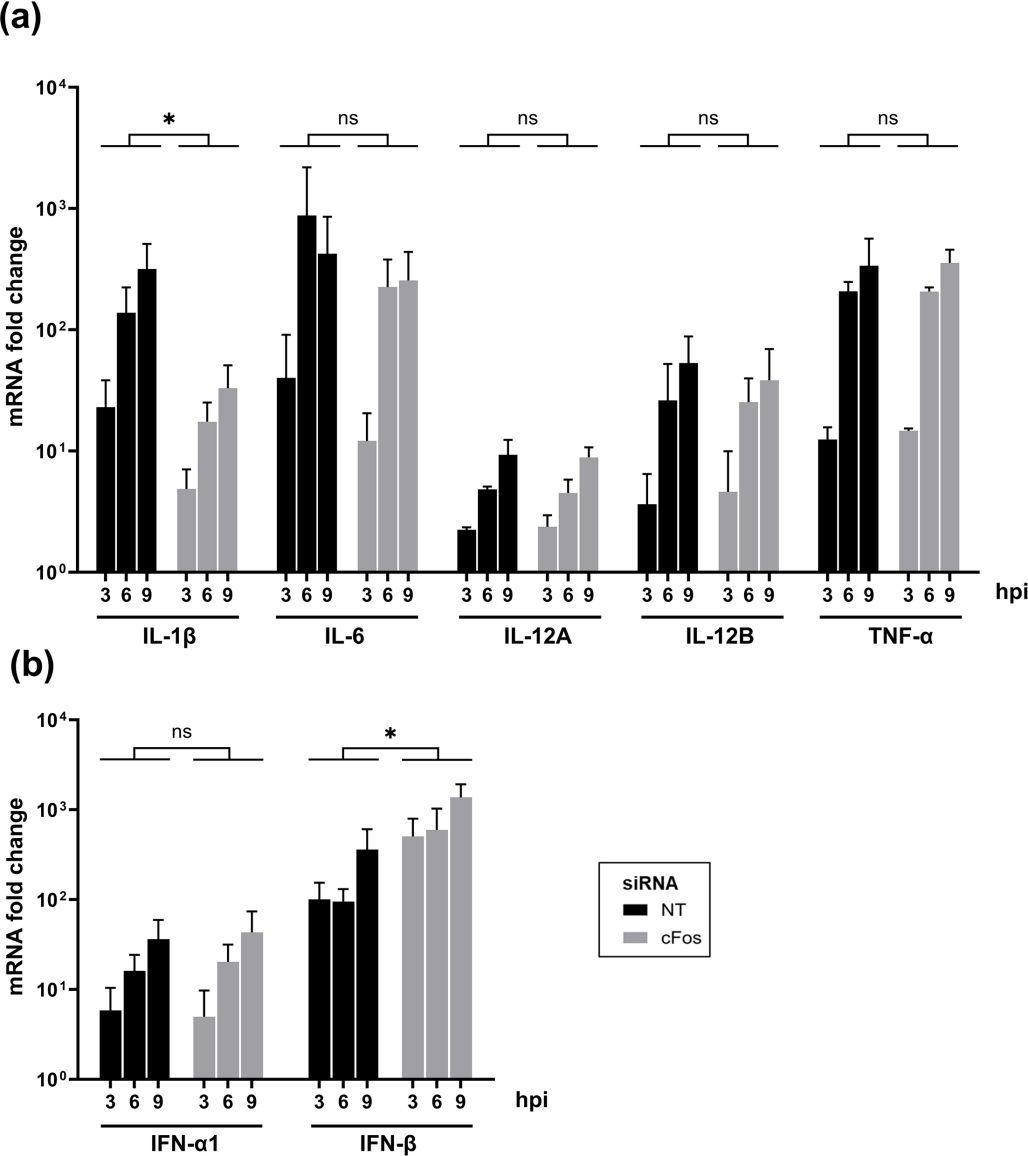
Inflammation and innate immunity are modulated in cFos–knockdown cells during IAV infection. A549 cells were treated with 25 nM of the NT or cFos siRNAs and infected 48 hpt with WSN at a moi of 3 p.f.u. cell^−1^. Total RNAs were extracted at indicated time points and subjected to real-time qPCR specific to (**a**) inflammatory cytokines IL-1*β*, IL-6, IL-12 (IL-12A and IL-12B) and TNF-*α* and (**b**) type I interferons IFN-*α*1 and IFN-*β*. mRNA fold changes relative to the condition 0 hpi were calculated using the 2^-ΔΔCt^ method. Results are expressed as the mean±sd of three independent experiments. The area under the curve (AUC) (not shown) was determined for each cytokine and siRNA condition in each independent experiment. The significance of the difference to NT was tested on AUCs with unpaired t-tests using GraphPad Prism software (ns, not significant, *P<0.05).

To monitor the regulation of cytokine expression by cFos, the expression of inflammatory cytokines IL-1β, IL-6, IL-12, TNF-*α* (Fig. 4a) and type I interferons IFN-*α*1 and IFN-*β* (Fig. 4b) was measured in cFos–knockdown cells during single-cycle IAV infection. NT-siRNA treatment was used as a control representing what happens upon IAV infection when endogenous cFos is unaffected. IL-1*β* mRNA induction was significantly lower in cFos–knockdown cells compared to the NT siRNA-treated cells (Fig. 4a). A decrease in the IL-6 mRNA induction, especially at 3 hpi, was also observed, but the overall difference was not statistically significant (Fig. 4a). No differences in IL-12A and IL-12B mRNA (both subunits forming the IL-12) as well as TNF-α mRNA were observed between cFos-knockdown and NT siRNA-treated cells (Fig. 4a). On the contrary, the mRNA level of IFN-*β* but not of IFN-*α*1 was significantly higher in cFos–knockdown cells (Fig. 4b). These findings suggest that, during IAV infection, cFos may promote the transcription of IL-1*β* and potentially IL-6 and repress that of IFN-*β*.

### Viral transcription and expression of viral proteins are impaired in cFos–knockdown cells

**Fig. 5.**
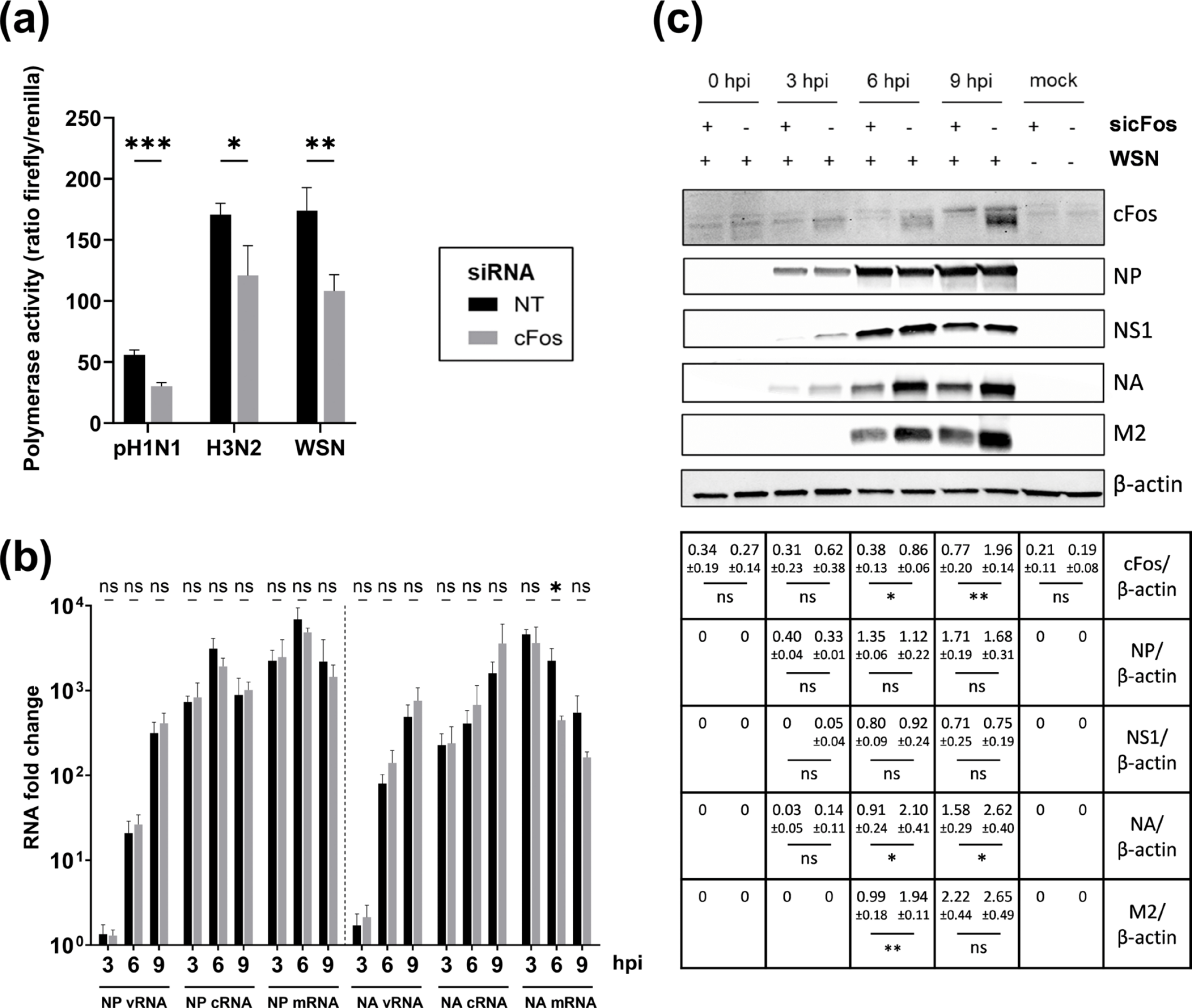
Viral transcription is impaired in cFos–knockdown cells. (**a**) HEK293T cells treated for 48 h with 25 nM of non-treated NT or cFos siRNAs were transfected with minigenome components. Firefly and *Renilla* luciferase activities were measured 24 hpt using the Dual-Glo Luciferase assay. Firefly luciferase activity (proportional to viral polymerase activity) was normalized to *Renilla* luciferase activity to take into account the transfection rate and cell density. Results are expressed as the mean ratio±sd of three independent experiments. The significance of the difference to NT was tested by an unpaired t-test in GraphPad Prism Software (*P<0.05, **P<0.01, ***P<0.001). (**b, c**) A549 cells were treated with 25 nM of NT or cFos siRNAs and infected 48 hpt with WSN at a moi of 3 p.f.u. cell^−1^. (**b**) Total RNAs were extracted at the indicated time points and subjected to strand-specific real-time qPCR [32]. RNA fold changes relative to the condition 0 hpi were calculated using the 2^-ΔΔCt^ method. The results are expressed as the mean±sd of three independent experiments. The significance of the difference to NT was tested with unpaired t-tests using GraphPad Prism software (ns, non-significant, *P<0.05). (**c**) Total cell lysates were harvested at the indicated times post-infection and analysed by immunoblot using antibodies directed against the indicated proteins. Immunoblot results representative of three independent experiments are shown. Band intensity of the indicated proteins was normalized to *β*-actin, and the mean ratios±sd of three independent experiments are presented in the table. The significance of the difference to NT (indicated by ‘-‘ in sicFos) was tested by unpaired t-tests in GraphPad Prism Software (ns, non-significant; *P<0.05, **P<0.01).

The consequences of cFos–knockdown on the viral replication were further evaluated. Minigenome assays for three human IAVs – pH1N1, H3N2 and WSN – showed a significant reduction of the viral polymerase activity in cFos–knockdown cells (Fig. 5a). The levels of vRNA, cRNA and mRNA production of NP and NA segments during single-cycle WSN infection were further evaluated in both cFos- and NT siRNA-treated cells using strand-specific real-time qPCR [32] (Fig. 5b). The production of NA mRNA was lower at 6 hpi (446 vs. 2,246 RNA fold change, *P*-value=0.023) and 9 hpi (162 vs. 548 RNA fold change, *P*-value=0.104) in cFos–knockdown cells compared to NT siRNA-treated cells. On the contrary, the production of vRNA and cRNA of NA tended to be slightly higher at 6 and 9 hpi, although the differences were not significant. For the NP segment, no significant differences were obtained, and the levels of mRNA, cRNA and vRNA did not appear to be affected by cFos–knockdown (Fig. 5b). Similar results were obtained at the protein level. Less NA and M2 viral proteins accumulated at 6 and 9 hpi in cFos–knockdown cells than in NT siRNA-treated cells, while no difference in the NP viral protein levels was observed. A slight decrease in NS1 viral protein seemed to occur at 3 hpi upon cFos–knockdown, but no such differences were observed at later time points. In agreement with what was observed at the mRNA level (Fig. 1a), cFos protein expression upon IAV infection was increased, especially at 9 hpi in control cells, and knockdown efficiency was confirmed in cFos-depleted cells as clearly observed at 6 and 9 hpi (Fig. 5c). The same pattern in the expression level of viral proteins was observed upon treatment with T-5224, the specific inhibitor of the cFos/AP-1 dimer DNA binding activity (Fig. S3b). Altogether, these findings highlighted a potential role of cFos in the viral transcription of lately expressed viral proteins.

## Discussion

AP-1 transcription factors are commonly induced by viral infections and were found to regulate the replication of several viruses [16,19]. IAV infection was shown to activate AP-1 transcription factors through phosphorylation by the JNK signalling pathway [24]. However, the role of AP-1 in IAV replication remains unclear. In the current study, we found that the transcription of cFos and cJun AP-1 factors was upregulated following IAV infection and that cFos appeared to support IAV replication.

The role of cFos is dependent on its location in the cell. At the endoplasmic reticulum, cFos activates *de novo* phosphatidylinositol phosphate (PIP) lipid synthesis by interacting with CDS1 and PI4KIIα enzymes [15]. The individual siRNA-based knockdown of CDS1 and PI4KIIα enzymes did not impair IAV replication (Fig. 2a). Although the individual knockdown of CDS1 in cardiomyoblast cells was previously shown to be critical and to impair the PIP pathway [36], it cannot be ruled out that CDS1 and PI4KII*α* protein isoforms might have played a compensatory role in our experiments. However, it is more likely that cFos does not influence IAV replication through its cytoplasmic role as an activator of PIP lipid synthesis, but rather through its nuclear role as an AP-1 transcription factor. This is further supported by the localization of cFos in the nucleoplasm during IAV infection (Figs 2c and S4) and the reduction of IAV replication in the presence of cFos nuclear activity inhibitor T-5224 (Fig. 2b). The most described cFos AP-1 dimer nuclear partner, cJun, did not appear to regulate IAV replication in our study, although it was previously found to support H5N1 IAV replication in A549 cells [23]. These discrepancies could be attributed to differences in the viral strains but more probably to the presence of residual cJun, since in our study, cJun depletion was less effective compared to cFos depletion. cFos might also regulate viral replication independently of its partner cJun, through binding to nucleic acids. In this regard, the overexpression of cFos mRNA upon infection may increase the likelihood of cFos forming homodimers or remaining as monomers, which were both shown to be able to bind cellular DNA [37,38]. The activity of cFos is regulated via phosphorylation by MAPK kinases, including ERKs and p38 [39,40]. These kinases are activated during IAV infection [41,42]. ERK5 was shown to phosphorylate cFos at the serine 32 position to increase its stability and nuclear localization [43]. Unfortunately, our experimental attempts using commercial antibodies did not allow us to distinguish the phosphorylated (Ser-32) from total cFos so far and, therefore, to determine whether cFos phosphorylation was necessary for the observed effect on the virus replication.

The role of cFos on apoptosis is unclear, with both pro- and anti-apoptotic roles mainly observed in cancer models [7,9, 44]. IAV was shown to protect cells from premature apoptosis [45]. However, at the late stage of infection, IAV promotes apoptosis to facilitate the spread of viral particles to neighbouring cells [45]. In our study, cFos–knockdown significantly increased apoptosis in IAV-infected A549 cells (Fig. 3). A similar increase in apoptosis was also observed in cFos–knockdown H1299 human epithelial lung cells infected with a gamma coronavirus [19]. Therefore, during viral infection, cFos seems to be involved in cell survival by inhibiting apoptosis.

Although certain transcription factors with known antiviral functions, such as STAT-1 and NF-κB p65, have been shown to facilitate IAV replication [46,47], the underlying molecular mechanisms remain poorly understood. AP-1 factors are widely recognized as transcriptional activators that promote expression of pro-inflammatory cytokines (reviewed in ref. [2]) and interferon-*β* and -*γ* [48,49]. Our results suggest that cFos may counteract cellular antiviral response by down-regulating virus-induced IFN-*β* expression (Fig. 4b). Activation of the *IFNb1* gene transcription occurs through a signalling cascade that requires the cooperative binding of ATF2/cJun AP-1 dimer [48]. Since cFos possesses a higher affinity for cJun than ATF2 and was shown to displace the ATF2/cJun dimer [50], cFos overexpression upon IAV infection could decrease cJun availability for ATF2, leading to a reduction in IFN-*β* transcription. The absence of the IFN-*β* expression or the inhibition of IFN-*β* activation by the cellular exonuclease XRN1 was shown to facilitate IAV replication [51,52]. IFN-*β* triggers the transcriptional activation of antiviral interferon-stimulated genes (ISGs). Given that these ISGs were shown to be upregulated at the early stage (4 hpi) during IAV infection in epithelial cells [53], enhanced ISG expression resulting from increased IFN-*β* levels could potentially account for the impaired viral transcription of NA mRNA observed in cFos–knockdown cells during single-cycle IAV infection experiments (Fig. 5).

The impaired viral transcription of NA mRNA (Fig. 5) may also suggest a direct role of cFos in the viral transcription. IAV relies on cap-snatching to obtain host-capped RNA fragments to initiate its own transcription, a process mediated by the interaction between the viral polymerase complex and the cellular DNA-dependent RNA polymerase II (RNAP II) [54]. cFos/cJun AP-1 dimer was shown to bind enhancer sequences and then to recruit the chromatin remodelling BRG1-associated factor (BAF) complex, leading to an accessible chromatin state [55]. Interaction between the Brg1 subunit of the BAF complex and the RNAP II was further shown to participate in the formation of the transcriptional pre-initiation complex [56]. As a transcription factor, cFos may thus facilitate the accessibility to the RNAP II for the viral polymerase complex, therefore helping in the cap-snatching process. Another chromatin remodelling complex was shown to support IAV viral transcription through direct interaction with the viral polymerase complex [57]. Using a split luciferase protein complementation assay, no direct interaction between cFos and any of the non-membranous viral proteins (Fig. S5a) or the viral polymerase proteins as a complex (Fig. S5b) could be found, indicating that cFos would most likely act through interaction with one or several additional cellular partners. Both IAV viral transcription and replication take place in the nucleus, and a conformational change of the viral polymerase is required to switch from transcription to replication status [58]. Interestingly, in parallel to the decrease of NA mRNA levels in cFos–knockdown cells, an increase of NA vRNA and cRNA levels seemed to occur, suggesting that cFos could be involved in the regulation of the timing of the viral transcription/replication switch.

In conclusion, this study highlighted cFos as a pro-viral factor that facilitates IAV replication. cFos was observed to extend cell survival and down-regulate IFN-*β* expression during IAV infection, potentially promoting viral replication. In addition, cFos may be mechanistically involved in viral transcription. However, the exact mechanisms by which cFos supports IAV replication remain unclear. It also remains enigmatic why the decrease observed in the transcription and expression of viral proteins only affected some viral proteins, especially those that are later expressed in the virus cycle.

## Supplementary material

10.1099/jgv.0.002194 Uncited Supplementary Material 1.

## Abbreviations

AP-1: activating protein-1
BAF: BRG1-associated factor
CDS1: CDP diacylglycerol synthase 1
DMEM: Dulbecco’s modified eagle’s medium
ER: endoplasmic reticulum
hpi: hours post-infection
hpt: hours post-transfection
IAV: influenza A virus
ISGs: interferon-stimulated genes
moi: multiplicity of infection
NP: nucleoproteins
NT: non-target
PI4KII*α*: phosphatidylinositol 4 kinase type II*α*
PIP: polyphosphoinositide
qPCR: quantitative PCR
RNAP II: RNA polymerase II
siRNA: small interfering RNA
vRNA: viral RNA
vRNP: viral ribonucleoprotein
