# Supplementary material for: The cellular activating protein-1 cFos regulates influenza A virus replication
Source: J Gen Virol. 2026 Jan 14;107(1):002194. doi: 10.1099/jgv.0.002194 (PMC12804346; doi:10.1099/jgv.0.002194)
Supplement: Uncited Supplementary Material 1. [file jgv-107-02194-s001.pdf]

Supplementary information

Table S1. siRNA sequences

siRNA	Sequences (5’-3’)
Non-targeting (NT) pool	UGGUUUACAUGUCGACUAA
	UGGUUUACAUGUUGUGUGA
	UGGUUUACAUGUUUUCUGA
	UGGUUUACAUGUUUUCCUA
cFos pool	GGGAUAGCCUCUCUUACUA
	ACAGUUAUCUCCAGAAGAA
	GAACCUGUCAAGAGCAUCA
	GCAAUGAGCCUUCCUCUGA
cJun pool	GAGCGGACCUUAUGGCUAC
	GAACAGGUGGCACAGCUUA
	GAAACGACCUUCUAUGACG
	UGAAAGCUGAGAACUCGGA
PI4KIIα pool	GGUUGGUGGUGCUGGAUUA
	CAACACUGAUCGAGGCAAU
	GAGACGAGCCCACUAGUGU
	GCAUCGGGCUACCACCAAA
CDS1 pool	AUUCUGAUAUUCCGGAAAU
	AUGAGUAAGUUGACGAUGU
	GAUGUAAACUCCUUCGUGA
	CAACCUGAACAGCAGUUAA
CDIPT pool	GUCACAAGAUGAUCGACUU
	GCCAAGAAGAAGUGACGCU
	GGCAUAUAGUAGCUGCUUA
	CACCUUGUGUGCUGGGAAU
RAB11A pool	GCAACAAUGUGGUUCCUAU
	CAAGAGCGAUAUCGAGCUA
	GUGCAGUGCUGUCAGAACA
	GAGAUUUACCGCAUUGUUU
PLK1 pool	GCACAUACCGCCUGAGUCU
	CCACCAAGGUUUUCGAUUG
	GCUCUUCAAUGACUCAACA
	UCUCAAGGCCUCCUAAUAG

Sequences designed by Horizon Discovery (commercially available).

Table S2. RT-qPCR primer sequences

Gene	Primer	Sequences (5'-3')
β-actin	Forward	AGGATTCCTATGTGGGCGAC
	Reverse	ATAGCACAGCCTGGATAGCAA
cFos [a]	Forward	GGGGCAAGGTGGAACAGTTAT
	Reverse	CCGCTTGGAGTGTATCAGTCA
FosB [a]	Forward	GCTGCAAGATCCCCTACGAAG
	Reverse	ACGAAGAAGTGTACGAAGGGTT
cJun [a]	Forward	AACAGGTGGCACAGCTTAAAC
	Reverse	CAACTGCTGCGTTAGCATGAG
JunB [a]	Forward	ACAAACTCCTGAAACCGAGCC
	Reverse	CGAGCCCTGACCAGAAAAGTA
JunD [a]	Forward	TCATCATCCAGTCCAACGGG
	Reverse	TTCTGCTTGTGTAAATCCTCCAG
Fra1 [a]	Forward	GGAGGAAGGAACTGACCGACTT
	Reverse	CTCTAGGCGCTCCTTCTGCTTC
Fra2 [a]	Forward	AAGAGGAGGAGAAGCGTCGCAT
	Reverse	GCTCAGCAATCTCCTTCTGCAG
ATF2 [a]	Forward	GGTAGCGGATTGGTTAGGACTC
	Reverse	TGCTCTTCTCCGACGACCACTT
IL-1β	Forward	CTAAACAGATGAAGTGCTCC
	Reverse	GGTCATTCTCCTGGAAGG
IL-6	Forward	GCAGAAAAAGGCAAAGAATC
	Reverse	CTACATTTGCCGAAGAGC
IL-12A	Forward	CCAGAAGGCCAGACAAACTC
	Reverse	GCCAGGCAACTCCCATTAG
IL-12B	Forward	GCGGAGCTGCTACACTCTC
	Reverse	CCATGACCTCAATGGGCAGAC
IFN-α1	Forward	ATCTGGTCCAACATGAAAAC
	Reverse	GGGTGAGAGTCTTTGAAATG
IFN-β	Forward	ATTCTAACTGCAACCTTTCG
	Reverse	GTTGTAGCTCATGGAAAGAG
CDIPT	Forward	AGGCAGTGAGAGTCACAAG
	Reverse	ACAGGTAGAGGAGGCAGTAG
CDS1	Forward	AGCACCAGCGACAAAGAAACAG
	Reverse	GCCCAGAACAAGAAGCATCAGC

PI4KIIα	Forward	TTTAACCGCATCGGGCTACCAC
	Reverse	TAACAGGCTCCTTCACCACCAC

[a] Sequences from: Yuan, L. X., Liang, J. Q., Zhu, Q. C., et al. 2021. A Gammacoronavirus, avian infectious bronchitis virus, and an Alphacoronavirus, porcine epidemic diarrhea virus, exploit a cell survival strategy by upregulating cFOS to promote virus replication. *Journal of Virology* 95 (4): e02107-20. https://doi.org/10.1128/JVI.02107-20.

Other sequences designed by Sigma-Aldrich (commercially available).

Table S3. Strand-specific RT-qPCR primer sequences (5'-3')

**Segment 5**			
vRNA	RT	vRNAtag WSNseg5 740F	GGCCGTCATGGTGGCGAATGAATGGACGGAGAACAAGGATTGC
	qPCR	vRNAtag	GGCCGTCATGGTGGCGAAT
		WSNseg5 845R	CTCAATATGAGTGCAGACCGTGCT
cRNA	RT	cRNAtag WSNseg5 1565R	GCTAGCTTCAGCTAGGCATCAGTAGAAACAAGGGTATTTTTCTTT
	qPCR	cRNAtag	GCTAGCTTCAGCTAGGCATC
		WSNseg5 1466F	CGATCGTGCCCTCCTTTG
mRNA	RT	mRNAtag WSNseg5 dTR	CCAGATCGTTCGAGTCGTTTTTTTTTTTTTTTTTCTTTAATTGTC
	qPCR	mRNAtag	CCAGATCGTTCGAGTCGT
		WSNseg5 1466F	CGATCGTGCCCTCCTTTG
**Segment 6**			
vRNA	RT	vRNAtag WSNseg6 689F	GGCCGTCATGGTGGCGAATACCATAATGACCGATGGCCCAAGT
	qPCR	vRNAtag	GGCCGTCATGGTGGCGAAT
		WSNseg6 839R	ACATCACTTTGCCGGTATCAGGGT
cRNA	RT	cRNAtag WSNseg6 1413R	GCTAGCTTCAGCTAGGCATCAGTAGAAACAAGGAGTTTTTTGAAC
	qPCR	cRNAtag	GCTAGCTTCAGCTAGGCATC
		WSNseg6 1314F	TGAATAGTGATACTGTAGATTGGTCT
mRNA	RT	mRNAtag WSNseg6 dTR	CCAGATCGTTCGAGTCGTTTTTTTTTTTTTTTTTTGAACAAACTAC
	qPCR	mRNAtag	CCAGATCGTTCGAGTCGT
		WSNseg6 1314F	TGAATAGTGATACTGTAGATTGGTCT

Sequences and RT-qPCR conditions from: Kawakami, E., Watanabe, T., Fujii, K., et al. 2011. Strand-specific real-time RT-PCR for distinguishing influenza vRNA, cRNA, and mRNA. *Journal of Virological Methods* 173 (1): 1-6. https://doi.org/10.1016/j.jviromet.2010.12.014.

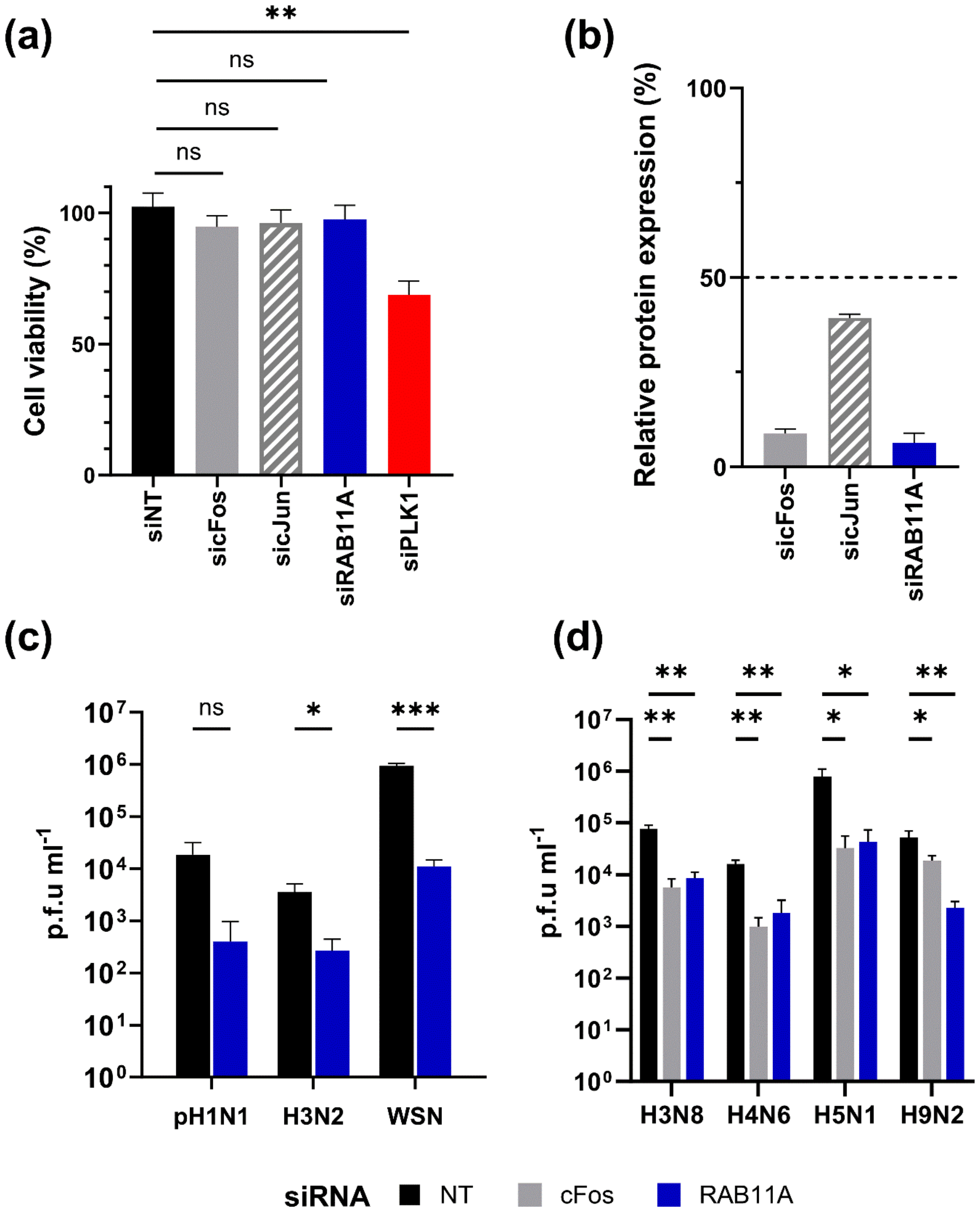

Fig. S1. Cell viability (a) and knockdown efficiency (b) upon siRNA transfection. (c) Control RAB11A siRNA treatment reduced IAV replication (d) cFos knockdown reduced IAV replication of avian IAVs. A549 cells were transfected with 25 nM of the NT or the indicated siRNAs. **(a)** Cell viability was determined at 48 hpt using the CellTiter-Glo Luminescent Viability Assay. Luciferase activities in the indicated siRNAs were compared to

the non-target (NT) treated cells to determine a percentage of viability. siPLK1 was included as a known cytotoxic control. The results are expressed as the mean percentages ± SD of three independent experiments. The significance of the difference to NT was tested with unpaired t tests using GraphPad Prism software (ns: non-significant, **P< 0.01). **(b)** At 24h post siRNA transfection, a second transfection with plasmids encoding cFos, cJun and RAB11A proteins fused with the full-length Gaussia luciferase (pGlucFL) was performed to assess knockdown efficiency. Ratios of the luciferase activities obtained in cells transfected with the indicated siRNAs to the ones obtained in cells transfected with the NT siRNA are shown. The results are expressed as the mean percentages ± SD of three independent experiments. The dashed line corresponds to a relative protein expression of 50% in silenced cells compared to NT-treated cells. **(c)** At 48 hpt, cells were infected with the following viruses at the indicated moi in p.f.u. $cell^{-1}$: pH1N1, moi of 10^{-3}; H3N2, moi of 10^{-2}; WSN, moi of 10^{-4}. At 24 hpi, viral titers were determined by plaque-forming assay. Results are expressed as the mean ± SD p.f.u. ml^{-1} of three independent experiments. The significance of the difference to NT was tested with unpaired t tests using GraphPad Prism software (ns: non-significant, *P< 0.05, ***P< 0.001). **(d)** At 48 hpt, cells were infected with the following viruses at the indicated moi in p.f.u. $cell^{-1}$: H3N8, moi of 10^{-1}; H4N6, moi of 10^{-2}; H5N1, moi of 10^{-2}; H9N2, moi of 10^{-1}. At 24 hpi, viral titers were determined by plaque-forming assay. Results are expressed as the mean ± SD p.f.u. ml^{-1} of three independent experiments. The significance of the difference to NT was tested with unpaired t tests using GraphPad Prism software (ns: non-significant, *P< 0.05, **P< 0.01).

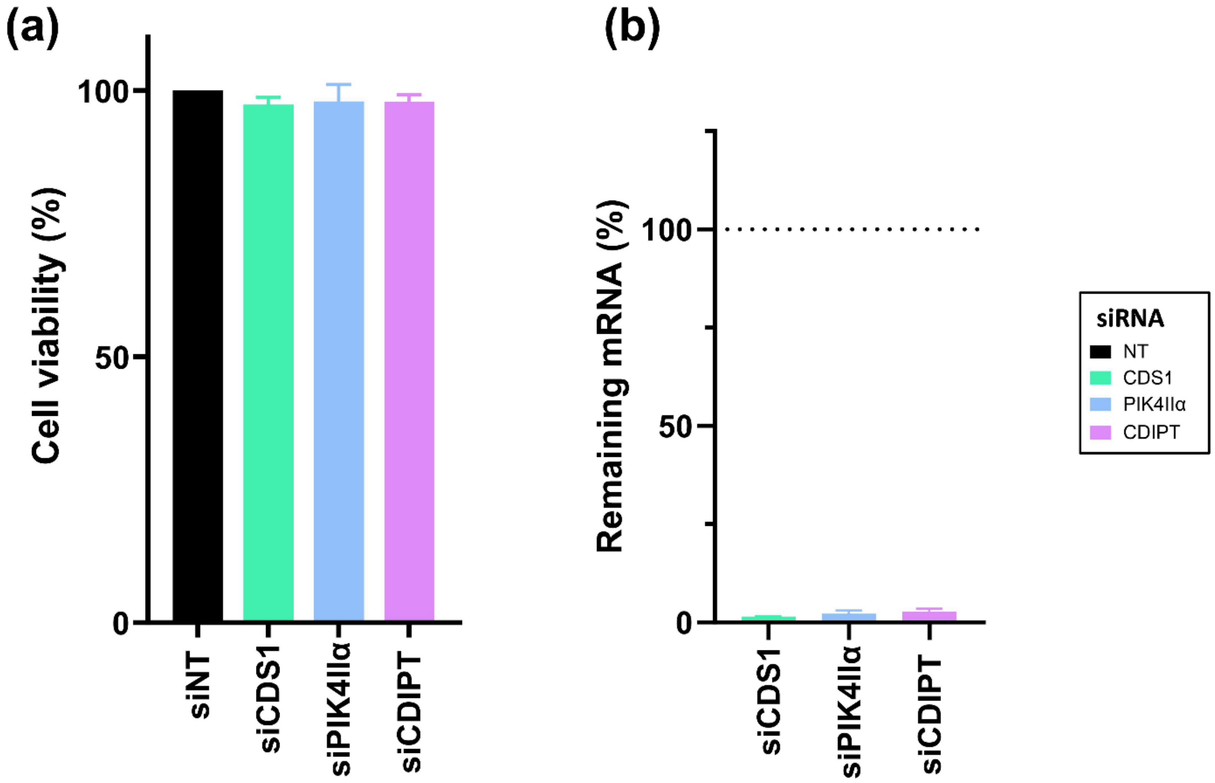

Fig. S2. Cell viability and knockdown efficiency upon siRNA knockdown of lipid synthesis enzymes. A549 cells were transfected with 25 nM of the indicated siRNAs **(a)** Cell viability was determined at 48 hpt using the CellTiter-Glo Luminescent Viability Assay. Luciferase activities in the indicated siRNAs and the non-target (NT) treated cells were compared to determine a percentage of viability. Results are expressed as the mean percentages ± SD of three independent experiments. **(b)** Total RNAs were extracted at 48 hpt and the mRNA expression level of each gene was determined by real time qPCR. Results are expressed as the mean percentages ± SD of remaining mRNA in each siRNA condition, as compared to NT control (calculated using the $2^{-\Delta\Delta Ct}$ method), determined in three independent experiments.

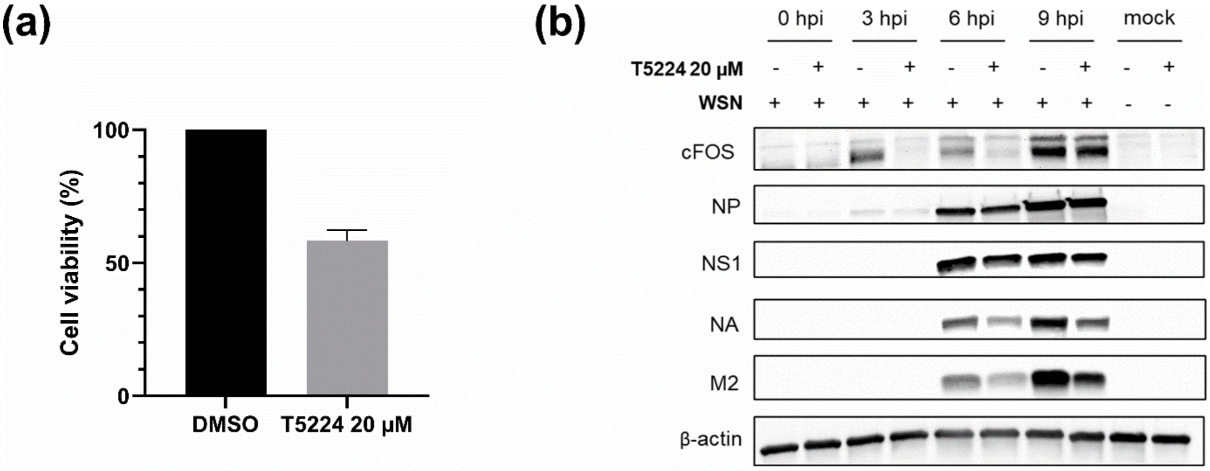

	0 hpi	3 hpi	6 hpi	9 hpi	mock
cFos/ β-actin	0.34 ±0.21 / 0.33 ±0.21 — ns	0.42 ±0.31 / 0.08 ±0.03 — ns	0.44 ±0.26 / 0.20 ±0.10 — ns	1.83 ±0.11 / 1.81 ±0.45 — ns	0.05 ±0.03 / 0.06 ±0.03 — ns
NP/ β-actin	0.04 ±0.03 / 0.05 ±0.04 — ns	0.21 ±0.14 / 0.30 ±0.27 — ns	2.04 ±0.85 / 1.78 ±0.66 — ns	2.37 ±0.47 / 2.03 ±0.29 — ns	0 / 0
NS1/ β-actin	0 / 0	0 / 0	2.00 ±0.33 / 1.63 ±0.15	2.17 ±0.06 / 1.51 ±0.09 — ***	0 / 0
NA/ β-actin	0 / 0	0 / 0	1.39 ±0,12 / 0.65 ±0,06 — **	2.07 ±0.14 / 1.31 ±0.13 — **	0 / 0
M2/ β-actin	0 / 0	0 / 0	1.86 ±0.37 / 1.26 ±0.31 — ns	3.65 ±0.42 / 2.58 ±031 — *	0 / 0

Fig. S3. Inhibition of AP-1 transcriptional activity of c-Fos/cJun AP-1 by the T-5224 inhibitor impaired viral protein expression. A549 cells were treated with DMSO or 20 µM T5224. **(a)** Cell viability was determined at 24 h post-treatment using the CellTiter-Glo Luminescent Viability Assay. Luciferase activities in the T-5224 condition were compared to the DMSO condition to determine a percentage of viability. The results are expressed as the mean percentages ± SD of three independent experiments. **(b)** At 16 h post-treatment, cells were infected with WSN at moi of 3 p.f.u. cell^{-1} in presence of T-5224 20µM or DMSO. Total cell lysates were harvested at the indicated times post-infection and analysed by immunoblot using antibodies directed against the indicated proteins. Band intensity of the indicated proteins was normalized to ß-actin and the mean ratios ± SD of three independent experiments are presented in the table. The significance of the difference to DMSO (indicated as '-' in T-5224) was tested by an unpaired t-test in GraphPad Prism Software (ns: non-significant, *P< 0.05, **P< 0.01, ***P<0.001).

(a)

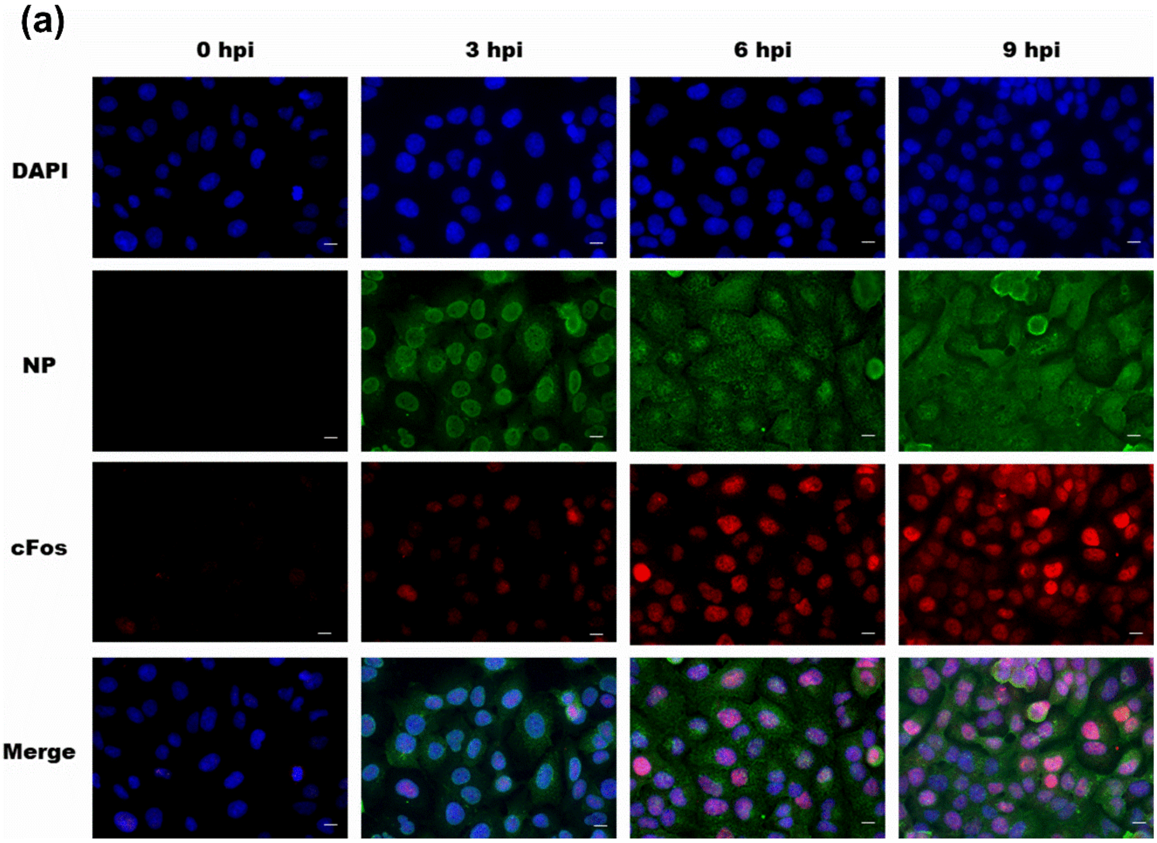

(b)

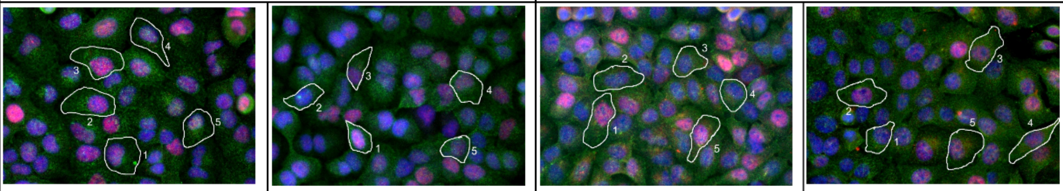

	Fibrillarin		Calreticulin	
	Replicate A	Replicate B	Replicate A	Replicate B
**Cell**	**% nuclear signal**	**% nuclear signal**	**% nuclear signal**	**% nuclear signal**
**1**	99.9%	99.5%	96.5%	89.2%
**2**	99.9%	98.9%	94.8%	98.7%
**3**	99.9%	99.9%	70.2%	86.5%
**4**	99.7%	100.0%	79.0%	87.2%
**5**	100.0%	100.0%	88.3%	85.9%
**Mean**	99.9%	99.7%	85.8%	89.5%
**SD**	0.1%	0.4%	9.9%	4.8%

Fig. S4. Immunofluorescence staining of cFos in A549 cells during single cycle infection (WSN, moi of 3 p.f.u. cell^{-1}). (a) Cells were fixed at 0, 3, 6 and 9 hpi, stained with DAPI and immuno-stained with anti-NP (infection control) and anti-cFos antibodies. Scale bar = 10 µm. **(b)** Quantification of cFos subcellular localization at 6 hpi. Since fibrillarin and calreticulin were detected using the same fluorophore, independent wells were used. In total, four independent infection wells were selected. For each image, five infected cells were analysed to

quantify the nuclear proportion of cFos. The red fluorescence signal corresponding to cFos was measured in the nucleus (defined by DAPI staining) and in the cytoplasm, using the Fiji software. The percentage of nuclear signal was calculated as the ratio of nuclear fluorescence to total cellular fluorescence (nuclear + cytoplasmic).

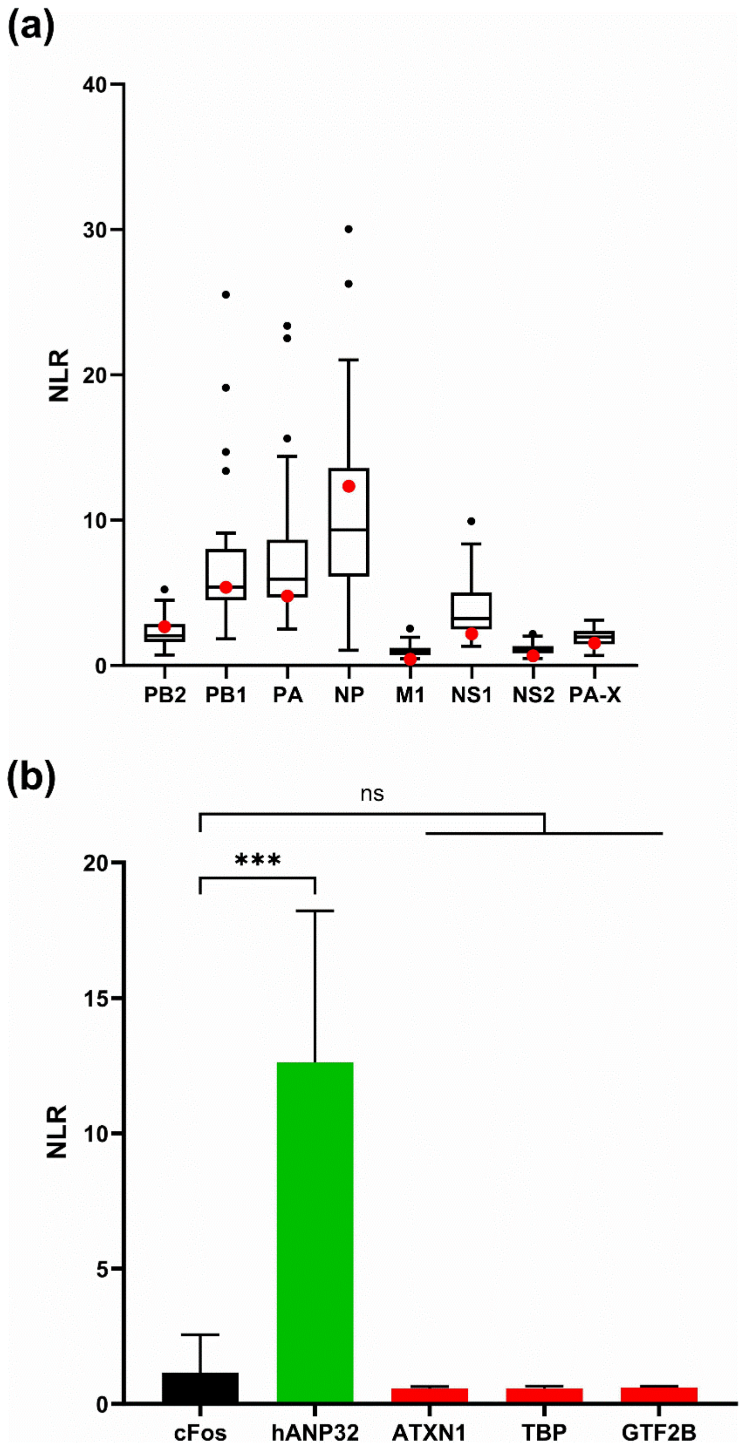

Fig. S5. Evaluation of the interactions between (a) IAV viral proteins and cFos, (b) IAV polymerase complex and cFos. (a) HEK293T cells were co-transfected with plasmids encoding a viral protein (WSN PB2, PB1, PA, NP, M1, NS1, NS2 or PA-X) fused to N2 split domain of the NanoLuc luciferase, and a cellular protein (cFos or one of 36 randomly selected proteins, used as negative controls), fused to the N1 split domain of the NanoLuc luciferase

(Choi, S.G., Olivet, J., Cassonnet, P. et al. 2019. Maximizing binary interactome mapping with a minimal number of assays. *Nat Commun* 10: 3907. https://doi.org/10.1038/s41467-019-11809-2). At 24 hpt, cells were lysed, and luminescence was measured (RLU). Normalized luminescence ratio (NLR) was calculated for each combination as follows: NLR = RLU (A-N1 + B-N2) / [RLU (A-N1 + N2) + RLU (N1 + B-N2)]. For each viral protein, the NLR distribution obtained with the randomly selected cellular proteins is represented as a Tukey box-and-whisker plot. A virus–host protein combination with an NLR value above the upper fence of the plot was considered as an interactor. NLR values obtained for cFos are indicated by red dots. **(b)** HEK293T cells were transfected with plasmids encoding WSN PB2 fused to Gluc1 split domain of the Gaussia Luciferase together with plasmids encoding WSN PA and PB1 (to reconstitute the viral polymerase), and cellular proteins (cFos, hANP32, ATXN1, TBP, or GTF2B) fused to Gluc2 split domain of the Gaussia Luciferase. At 24 hpt, cells were lysed, luminescence was measured (RLU), and NLR was calculated (Cassonnet, P., Rolloy, C., Neveu, G. et al. 2011. Benchmarking a luciferase complementation assay for detecting protein complexes. *Nat Methods* 8: 990–992. https://doi.org/10.1038/nmeth.1773). The human ANP32 (hANP32), described to interact with the polymerase complex (Sugiyama, K., Kawaguchi, A., Okuwaki, M., Nagata, K. 2015. pp32 and APRIL are host cell-derived regulators of influenza virus RNA synthesis from cRNA. *eLife* 4: e08939. https://doi.org/10.7554/eLife.08939), was considered as a positive control (green bar) while ATXN1, TBP, and GTF2B were randomly selected proteins (negative control - red bars). The results are expressed as the mean NLR ± SD of three independent experiments. The significance of the difference to cFos was tested by unpaired t-tests in GraphPad Prism Software (ns: non-significant, ***P< 0.001).
